# Strong antibody reactivity to HIV-1 synthetic peptides in seropositive indigenous Warao people

**DOI:** 10.7705/biomedica.7510

**Published:** 2025-05-30

**Authors:** Isabel Durango, Sandra Losada, Henry Bermúdez, Julián A. Villalba, Yoneira Sulbaran, Rossana C. Jaspe, Jacobus H. de Waard, Héctor R. Rangel, Óscar O. Noya, Flor H. Pujol

**Affiliations:** 1 Laboratorio de Virología Molecular, Centro de Microbiología y Biología Celular, Instituto Venezolano de Investigaciones Científicas, Caracas, Venezuela Instituto Venezolano de Investigaciones Científicas Instituto Venezolano de Investigaciones Científicas Caracas Venezuela; 2 Sección de Biohelmintiasis, Instituto de Medicina Tropical, Facultad de Medicina, Universidad Central de Venezuela, Caracas, Venezuela Universidad Central de Venezuela Universidad Central de Venezuela Caracas Venezuela; 3 Department of Pathology and Laboratory Medicine, Emory University School of Medicine, Atlanta, GA, USA Emory University School of Medicine Emory University School of Medicine Atlanta GA USA; 4 Departamento de Tuberculosis, Servicio Autónomo Instituto de Biomedicina "Dr. Jacinto Convit", Universidad Central de Venezuela, Caracas, Venezuela Universidad Central de Venezuela Universidad Central de Venezuela Caracas Venezuela

**Keywords:** HIV-1, peptides, antibodies, indigenous people, receptors, CXCR4, Venezuela, HIV-1, péptidos, anticuerpos, pueblos indígenas, receptores CXCR4, Venezuela

## Abstract

**Introduction.:**

Previous studies have described an epidemic of HIV-1 in the indigenous Warao population living in the Orinoco Delta, Venezuela. The Warao face extraordinary challenges amid of their ongoing HIV-1 epidemic, the highest reported HIV-1 prevalence in indigenous groups (9.6%) in South America.

**Objective.:**

To investigate the antibody reactivity to HIV-1 synthetic peptides in seropositive individuals, with a particular focus on the indigenous Warao population from Venezuela.

**Materials and methods.:**

The HIV-1 *Pol* region from infected patients’ isolates was amplified, sequenced, and analyzed using phylogenetic tools. Custom-designed synthetic peptides were derived from conserved regions of HIV-1 glycoproteins 41 and 120, based on reference sequences. Multiple antigen blot assays were used to evaluate the presence of antibodies against synthetic peptides.

**Results.:**

The most frequent HIV-1 subtype was B, the most common in Venezuela, although some individuals were infected with subtype A1. Distinct patterns of reactivity to synthetic peptides were observed between the sera of the general population and the Warao population; the sera of the latter exhibited a high intensity of peptide recognition.

**Conclusions.:**

The use of synthetic peptides, coupled with the robust performance of multiple antigen blot assays, enriches our understanding of antibody responses in different HIV-1-infected populations.

## Resumen

**Introducción.:**

Estudios anteriores han descrito una epidemia de HIV-1 en la población indígena warao que vive en el delta del Orinoco de Venezuela. La comunidad warao enfrenta desafíos extraordinarios para combatir esta epidemia que tiene la prevalencia más alta de HIV-1 reportada para grupos indígenas (9,6 %) en Suramérica.

**Objetivo.:**

Investigar la reactividad de los anticuerpos a los péptidos sintéticos de HIV-1 en personas seropositivas, con especial atención en aquellas de la población indígena warao de Venezuela.

**Materiales y métodos.:**

La región *Pol* del virus HIV-1 fue amplificada y secuenciada para el análisis filogenético de los aislamientos virales obtenidos de los pacientes seleccionados. Los péptidos sintéticos se diseñaron a partir de las regiones conservadas de las glucoproteínas 41 y 120 del HIV-1 de las secuencias de referencia. Se utilizó el ensayo de transferencia de antígenos múltiples para evaluar la presencia de anticuerpos contra los péptidos.

**Resultados.:**

El subtipo de HIV-1 más frecuente fue el B -el más común en Venezuela-, aunque algunos individuos estaban infectados con el subtipo A1. Se observaron distintos patrones de reactividad a los péptidos sintéticos en los sueros de la población general versus los de la comunidad warao; estos últimos reconocieron intensamente a los péptidos.

**Conclusiones.:**

El uso de péptidos sintéticos junto con la solidez del ensayo de transferencia de antígenos múltiples enriquece la comprensión de la respuesta inmunitaria mediada por anticuerpos en diferentes poblaciones infectadas por HIV-1.

Despite several advances in highly active antiretroviral treatment (HAART), human immunodeficiency virus (HIV) infection remains a serious public health problem, with an estimated 37 million people infected worldwide ^1^. Indigenous populations in South America living in isolated habitats are usually free of HIV infection yet they are particularly vulnerable when HIV-1 is introduced into their communities ^2^.

Previous studies have described an epidemic of HIV-1 in the indigenous Warao population living in Venezuela’s Orinoco Delta ^3^. The Warao, characterized by a particular cultural identity and a specific geographical area, face extraordinary challenges amid their ongoing HIV-1 epidemic, which has the highest HIV-1 prevalence in indigenous groups (9.6%) reported in South America ^2^^,^^3^. This epidemic is associated with a generally rapid progression to acquired immunodeficiency syndrome (AIDS).

The highest national incidence of tuberculosis is also found among the Warao people ^4^, and coinfection with tuberculosis is known as an adverse prognostic factor for the progression of HIV-1. In addition, an extremely high frequency of CXCR4-tropic (X4) HIV-1 strains was found among infected Warao individuals, suggesting either an initial infection dominated by X4- type strains or a very rapid selection of X4 variants after infection ^5^. The envelope protein of HIV-1 interacts with CD4 and CCR5 (R5) and/or CXCR4 co-receptors on the cell membrane. R5 viruses are selected during primary infection through sexual transmission. During the natural course of the infection, patients gradually switch to X4 virus strains, a transition associated with worsened clinical progression ^6^.

Synthetic peptides have been valuable tools for immunodiagnostic purposes for several pathogens ^7^. In indigenous populations, they have been used to detect *Trypanosoma cruzi* exposure ^8^, and to discriminate between HTLV-1 and HTLV-2 infections ^9^. Specifically, within the Warao community, synthetic peptides have allowed to differentiate between active and latent tuberculosis ^10^. It has been proven that these peptides are profitable instruments in the immunodiagnosis of HIV and are especially viable in differentiating between HIV-1 and HIV-2 infections ^11^. Building on this premise, we sought to utilize the potential of synthetic peptides to analyze the complex antibody responses in diverse Venezuelan populations living with HIV-1.

The aim of this study was to analyze the immune response to HIV synthetic peptides, specifically designed for this research, in different population groups from Venezuela, with a particular interest in the Warao community, who are facing a devastating HIV-1 epidemic.

## Materials and methods

### Synthetic peptides

We custom-designed synthetic peptides derived from conserved regions of the HIV-1 glycoproteins 41 and 120 (gp41, gp120), according to reference sequences:

- Peptides IMT1954 and IMT2197 were generated considering the HIV- 1 gp41 reference sequence of amino acids (AKZ15547). However, peptide IMT1954 has two additional glycine and cysteine (Cys-Gly--- Gly-Cys) at the 5' and 3'ends, whereas peptide IMT2197 has Cys-Gly addition only at the 5' end.
- Peptides IMT1961 and IMT1963 were created also based on the of the HIV-1 gp41 reference sequence (AKZ15547). However, peptide IMT1961 has an additional Cys-Gly---Gly-Cys at the 5' and 3', whereas peptide IMT1963 has a Gly-Cys addition at the 3' end.
- Peptides IMT1965 and IMT1968 were synthesized using the HIV-1 gp120 reference sequence of amino acids (ACN87096). However, peptide IMT1968 has an additional Cys-Gly---Gly-Cys at the 5' and 3'ends, whereas peptide IMT1963 has a Gly-Cys addition at the 3' end.

The addition of the two amino acids (Cys/Gly) is intended to promote polymerization, as previously described ^12^.

### Serum samples

The 110 blood plasma samples -positive and negative for HIV- 1- preserved at -70°C and used in this study were collected through venipuncture in previous years (general population, 2009-2018; Warao population, 2012). The Bioethics Commission of the *Instituto Venezolano de Investigaciones Científicas* approved patients’ informed consent and the study development. The positive samples were from Venezuelan individuals diagnosed with HIV-1 by enzyme-linked immunosorbent assay (ELISA) or rapid tests, most of whom had detectable HIV-1 RNA (except for some patients treated with antiretroviral drugs). Two of these samples were from long-term non-progressors (LTNP). For Warao individuals, blood samples were tested in the field with HIV-1/2 rapid assays (First Response^TM^ HIV 1-2-0 Card Test, Premier Medical Corporation Private Limited) (Gujarat, India). Positive patients were confirmed by ELISA and immunoblot. All Warao reactive samples were positive for HIV-1 viral RNA, as previously described ^3^. HIV-1-infected Warao individuals were treatment-naive and had no HIV-2 viral RNA detected in them. Control samples were taken from HIV-1-negative individuals, from both the Warao and the general population.

### Sequence analysis

The amplicons of the partial HIV-1 *Pol* genomic region were sequenced (nucleotides 2,057-3,587 relative to the HXB2 complete reference genome), as previously described ^13^.

### Serological assays

A multiple antigen blot assay (MABA) was performed with nitrocellulose strips coated with synthetic peptides ^14^^,^^15^. Coated nitrocellulose strips were incubated for 90 minutes at room temperature with human sera (1:100) in a blocking solution containing 5% skim milk in PBST pH = 7.5 (80 mM Na_2_HPO_4_, 20 mM NaH_2_PO4, 100 mM NaCl, and 0.05% Tween-20). Then, the samples were incubated overnight at 4°C with a secondary anti-human IgG goat antibody coupled with alkaline phosphatase (Sigma, catalog No. A-3150) and diluted (1:5,000) in the blocking solution. The precipitable chromogenic detection system SIGMAFAST BCIP/NBT tablets (Sigma, catalog No. B5655) was used as a substrate according to the manufacturer’s instructions.

### Statistical analysis

The frequency of peptide recognition between groups was compared using chi-square and EpiInfo 7.2.5.0 software ^16^. Significant differences in the number of bands recognized by the sera of each group were assessed using Student’s t-test.

## Results

**Figure 1:**
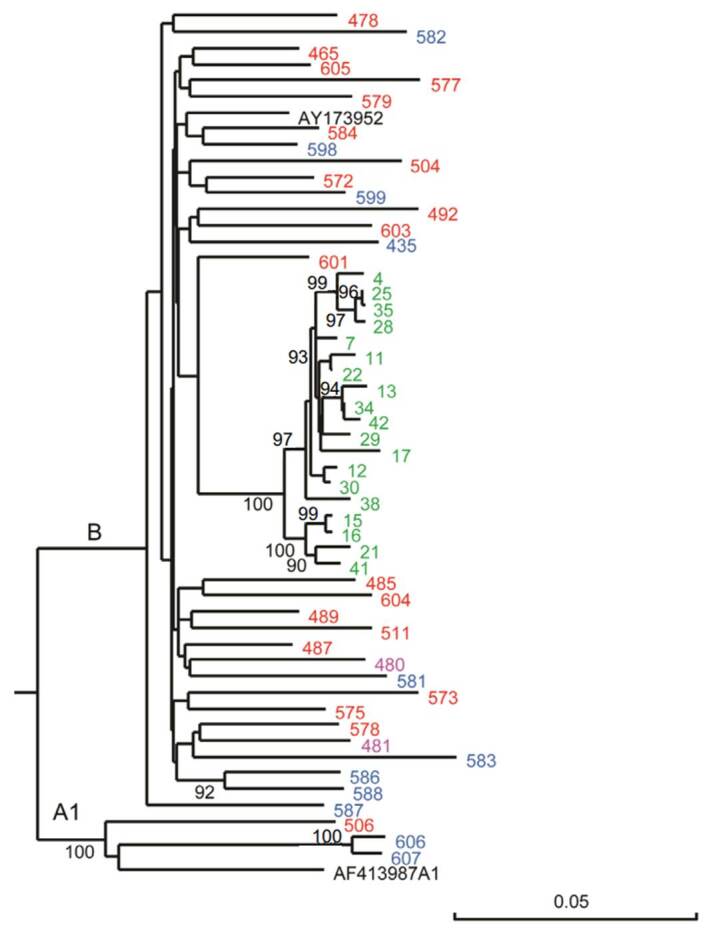
Maximum likelihood phylogenetic analysis of HIV-1 isolates from study participants. We analyzed approximately 1,200 nucleotides of the HIV Pol region using the HKY nucleotide substitution model with 500 bootstrap replicates to assess branch support. The tree shows HIV-1 sequences from the general population: Blue for treatment-naive individuals, pink for long-term non-progressors, and red for those undergoing treatment. The two reference sequences -representing known subtypes A1 and B- are shown in black. Subtype designations are indicated on the respective branches. The numbers per node refer to the bootstrap values exceeding 90%, signifying robust phylogenetic relationships. Some viral sequences from certain individuals were unavailable and therefore are not represented in this analysis.

We analysed 110 serum samples, but the HIV-1 *Pol* sequence was available only in 62 samples. The individuals tested in the multiple antigen blot assay resulted with infections caused by two different subtypes of HIV-1: 59 were subtype B, the most common in Venezuela ^13^, and 3 with subtype A1. All viral sequences infecting the Warao people formed a monophyletic group inside subtype B, a product of the infection of one individual who spread the virus to many others within the population, as previously described ^3^ (figure 1).

**Figure 2:**
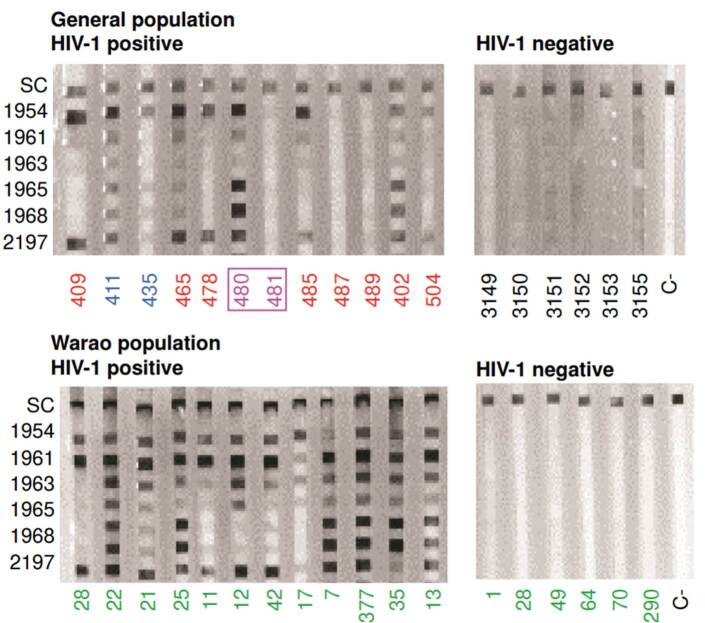
Multipleantigenblotassay(MABA)withHIVsyntheticpeptides.SixHIV-1peptides were tested corresponding to 3 HIV-1 amino acid sequences. The reactivity of some representative sera is shown. The MABA with sera from the general population is shown in blue (treatment-naive) or red (treated). Samples from Warao individuals are shown in green. Sera from samples shown in pink and enclosed in a box are from LTNPs. C- refers to the negative control without serum. In the y-axis, SC is the serum control, and the numbers refer to the IMT peptides.

As shown in table 1, most HIV-1-positive samples detected the peptides, and no reactivity was observed with the negative sera from the general population and the Warao people. The three HIV-1 subtype A1 samples recognized all the peptides tested (data not shown). Four HIV-1-positive samples did not react to the synthetic peptides: one of them was from a longterm non-progressor, and the other three were from treated patients receiving different treatments (table 1). The reactivity of two samples from long-term non-progressors was evaluated: one exhibited a strong reactivity to all synthetic peptides, while the other failed to recognize any (figure 2).

**Table 1:** Antibody reactivity to at least one of the three HIV synthetic peptides according to the multiple antigen blot assay

Population	Reactivity (at least 1 out of 3 peptides)
HIV-1 positive, treatment-naive	39/40 (98%)
General population	19/20 (95%)
Warao population	20/20 (100%)
HIV-1 positive, treated	52/55 (95%)
Total HIV-1 positive	91/95 (96%)
HIV-1 negative	
General population	0/8
Warao population	0/7

The reactivity to the three pairs of synthetic peptides, two derived from HIV-1 gp41 and one from gp120, was tested in this study. Each pair consists of a core peptide sequence, but one peptide has a Gly-Cys/Cys-Gly addition at one end (5’ or 3’), while the other has a Gly-Cys---Cys-Gly inserted at both ends (5’ and 3’). These additions aimed to promote peptide polymerization and higher immune reactivity. Different degrees of sample reactivity were observed, as shown in figure 2.

Reactivity to peptides with one pair of amino acids (Gly-Cys/Cys-Gly) at one end (5’ or 3’) was equal to or higher than that of peptides with two pairs of amino acids (Gly-Cys---Cys-Gly) (data not shown). Therefore, for reactivity analysis, we only considered the recognition of the three peptides modified with Gly-Cys or Cys-Gly.

**Figure 3:**
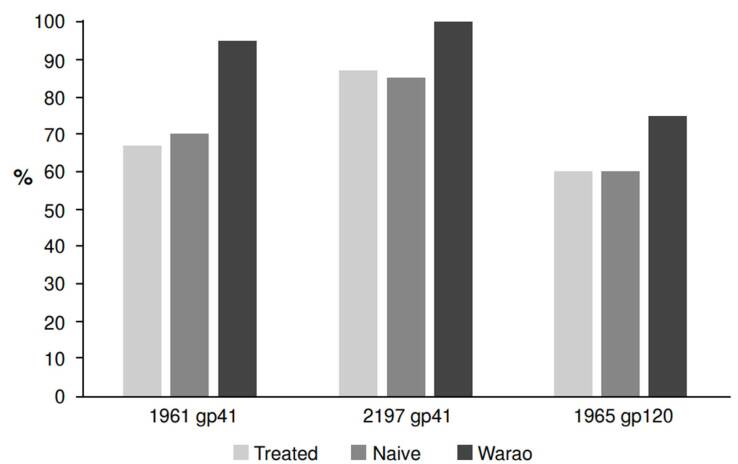
Frequency of recognition for each synthetic peptide. The reactivity to HIV-1 peptide IMT1963 was not considered for this analysis since it was less frequently recognized than peptide IMT1961.

Figure 3 shows the frequency of recognition per peptide in both groups. The most frequently recognized peptide was IMT2197 in the two groups. The frequency of peptide recognition was similar between treatment- naive individuals and treated patients from the general population. Higher recognition frequencies for the three included peptides were observed in the Warao group. Specifically, for the peptide IMT1961, the frequency of recognition between treated and treatment-naive HIV-1 patients from the general population was significantly lower than that observed for the Warao individuals (51/75, 68% versus 19/20, 95%, Fisher’s exact test = 0.01).

**Figure 4:**
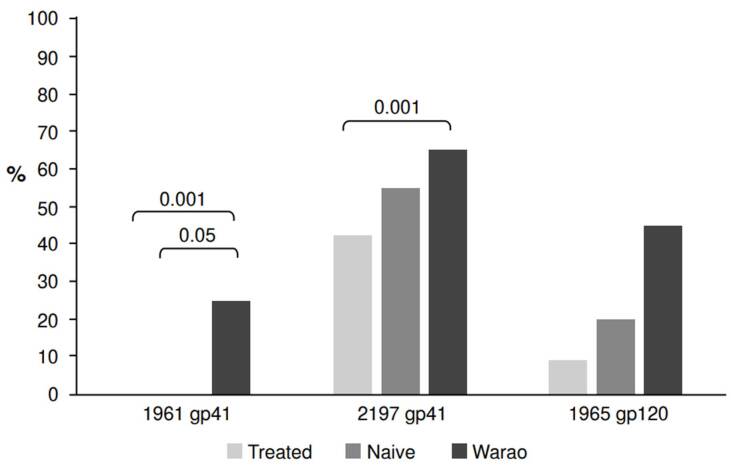
Frequency of recognition with an intense band for each synthetic peptide. Significant differences are shown above the brackets. The reactivity to HIV-1 peptide IMT1963 was not considered for this analysis since it was less frequently recognized than peptide IMT1961.

The frequency of high-intensity band recognition was also compared for each peptide (figure 4). A gradient in recognition was observed between samples from treated patients and treatment-naive HIV-1 individuals in both populations. Our study revealed a statistically significant difference in antibody reactivity patterns between samples from individuals in the general population under antiretroviral therapy and those from the Warao community for peptides 1961 and 2197.

## Discussion

The HIV-1 synthetic peptides designed for this study were recognized by most serum samples from the evaluated HIV-1-infected individuals in Venezuela. These peptides were detected primarily by HIV-1 subtype B isolates -by far the most common subtype circulating in the country ^13^- but also by subtype A1.

Distinct recognition patterns of HIV-1 synthetic peptides were found among different populations living with HIV-1 in Venezuela. Notably, individuals receiving antiretroviral therapy exhibited a lower frequency and intensity of peptide recognition. This attenuated response among treated patients is a well-documented phenomenon, also reflected in rapid tests. The intensity of the antibody response is related to the size of the HIV-1 reservoir, as antibody levels depend on the continuous stimulation by HIV-1 antigens. A recent meta-analysis indicated an average reduction of up to 16% in antibody reactivity in treated adults living with HIV-1 when antiretroviral treatment was initiated less than six months after diagnosis. In contrast, only a few seronegative cases were found when treatment was initiated later. The observed frequency of seroreversion was higher when using rapid tests ^17^.

Another interesting observation was the contrasting reactivity exhibited by two long-term non-progressor samples. The strong reactivity exhibited by one of the serums to all synthetic peptides suggested a robust and sustained immune response, potentially contributing to the non-progressive nature of the infection. Conversely, the other serum lacked recognition of these peptides, implying a wide range of immune profiles even within this subgroup. The host and/or virus may play a role in determining a long-term non-progressor phenotype after HIV-1 infection. Factors such as a vigorous innate and adaptive response or mutations in viral genes (particularly accessory ones) are involved in long-term non-progression to AIDS ^18^^-^^20^. We speculate that the first case of the long-term non-progressors may represent an immune-related phenotype characterized by a strong antibody response. In contrast, the second case may exemplify a viral-related phenotype, leading to reduced antibody reactivity due to low viral replication inherent to the viral strain. Analysis of the HIV-1 *Pol* sequences of both individuals did not reveal evidence of APOBEC3G activity, suggesting that the long-term non-progressor phenotype was not associated with a defect in the viral protein Vif ^18^.

Studies of the Warao indigenous population revealed a particular immunogenetic background not seen previously in other human populations ^21^, affecting IFN-γ production. This indigenous population exhibits the highest incidence rate of tuberculosis in Venezuela, probably related to the frequent presence of genes associated with susceptibility to *Mycobacterium tuberculosis*^22^. However, it does not necessarily imply a reduced antibody reactivity to specific antigens. An example of this scenario is the strong antibody response, particularly in Warao children with signs of malnutrition, against the pneumococcal vaccine ^23^.

Notably, the Warao population displayed higher recognition frequencies for the HIV-1 peptides developed in this study. This distinctive pattern suggests a unique immune response profile within the Warao group, potentially shaped by genetic, environmental, and epidemiological factors specific to this population. Moreover, the unique challenges posed by the HIV-1 epidemic within the Warao population, coupled with the CXCR4 viral strain characteristics ^5^, add a layer of complexity to our understanding. Contrary to our expectations and considering the rapid evolution of AIDS in the Warao community, the antibody response in this context was notably robust, challenging prevailing notions regarding responses in distinct populations.

The satisfactory performance of synthetic peptides in this study positions them as valuable tools for epidemiological research, offering a nuanced understanding of antibody responses in different HIV-1-infected populations ^11^. The application of synthetic peptides not only enhances diagnostic accuracy but also provides a foundation for personalized approaches to HIV care, emphasizing the importance of considering diverse immune profiles in the broader context of public health. The multiple antigen blot assay efficiently captured nuanced antibody reactivity patterns among cohorts. This technique effectively identified specific peptides that were particularly recognized, showcasing its potential for in-depth analysis of antibody response ^14^.

Our exploration of the Warao population during a significant HIV-1 epidemic caused by a unique CXCR4 viral strain revealed a surprisingly resilient antibody response. This scenario challenges preconceived notions regarding immune responses in unique subpopulations. The distinct antibody reactivity patterns observed within the Warao group underscore the importance of considering population-specific factors in HIV research.

It would be interesting to analyze the isotype of the IgG recognizing these peptides. In addition, the viral load was unavailable for each patient, particularly for the Warao community, so it was not possible to correlate the intensity of the peptide recognition with that important parameter.

Another limitation of this study is the relatively small number of samples tested, which did not allow us to reach significance in some observed patterns. However, the tendency of recognition for each group was as expected.

In summary, synthetic peptides coupled with the robust performance of the multiple antigen blot assay enriched our understanding of antibody responses in different HIV-1-infected populations. Warao individuals affected by this pathogen had a strong reactivity to the HIV-1 synthetic peptides. As we continue to unravel the complexities of HIV dynamics, these findings pave the way for more targeted and personalized approaches to diagnosis, treatment, and public health initiatives.
